# In Vitro Screening for Cytotoxic Activity of Herbal Extracts

**DOI:** 10.1155/2017/2675631

**Published:** 2017-03-13

**Authors:** Valter R. M. Lombardi, Iván Carrera, Ramón Cacabelos

**Affiliations:** Health Biotechnology Department, EBIOTEC, Bergondo, La Coruña, Spain

## Abstract

Experimental studies have shown that a variety of chemopreventive plant components affect tumor initiation, promotion, and progression and the main difference, between botanical medicines and synthetic drugs, resides in the presence of complex metabolite mixtures shown by botanical medicine which in turn exert their action on different levels and via different mechanisms. In the present study, we performed an in vitro screening of ethanol extracts from commercial plants in order to investigate potential antitumor activity against human tumor cell lines. Experimental results obtained through a variety of methods and techniques indicated that extracts of* I. verum*,* G. glabra*,* R. Frangula*, and* L. usitatissimum* present significant reduction in* in vitro* tumor cell proliferation, suggesting these extracts as possible chemotherapeutical adjuvants for different cancer treatments.

## 1. Introduction

The plant kingdom contains a great source of new bioactive compounds which, due to their intrinsic biological properties, may be used in medicine as well as in other human health promoting areas. Phytopharmaceuticals play an important role in general medical practice for the treatment of diseases of the cardiac and vascular system [1], nervous system [2], and immune system [3], and a large number of herbal drugs have a suggested prophylactic effect besides their use for therapy of diseases [4, 5]. It has been estimated that 7 to 10 years and a cost over $5,000,000 are required to develop new anticancer drugs. This includes costs for the initial collection of specimens, evaluation of crude extracts, purification, identification and synthesis of the active substance, and preclinical and clinical studies. Many commercially sold medicinal plants might contain chemical substances with potential mutagenic and/or carcinogenic properties [6, 7] as well as with antitumor properties [8, 9], and the active extracts detected by screening methods should be subjected to accurate bioassays to determine their specific pharmacological activity.

The increase in interest in apoptosis, or programmed cell death (PCD), has had a major impact on many fields within the biological sciences, including oncology [10, 11]. The delineation of discrete apoptotic pathways has affected not only our basic concepts of the development of cancer, but also our approaches toward the prevention and therapy of the disease. It is now evident that the balance between cellular proliferation and death plays a vital role in the maintenance of normal tissue homeostasis, and the derangements of either of these processes can lead to the dysregulated clonal expansion that is characteristic of all neoplastic diseases [12–15]. The recognition of apoptosis as a major mechanism of action of many standard cytotoxic agents has led to novel experimental approaches aimed at stimulating apoptotic pathways in order to improve therapeutic response. The essential role of natural compounds in regulating cellular proliferation and differentiation has been known for over 50 years, yet their importance as physiological and pharmacological regulators of cell death has only recently been appreciated. The recognition of natural products as prominent mediators of the critical pathways involved in the development and progression of cancer has renewed interest in their potential as chemopreventive and chemotherapeutic anticancer agents [16, 17].

In the present study, we report comparative data regarding the in vitro cytotoxic effect of different plant extracts on a panel of human tumor cell lines. Our results indicate that the* Illicium verum, Glycyrrhiza glabra, Rhamnus Frangula, *and* Linum usitatissimum *extracts induce downregulation of antiapoptosis genes at the same time that leads to a strong cytotoxic effect against the majority of tested tumor cells.

## 2. Methods

### 2.1. Materials

The human liposarcoma cell line (SW872), the human synovial sarcoma cell line (SW982), the human bone osteosarcoma cell line (HS 39.T), the human connective tissue leiomyosarcoma cell line (HS 5.T), the human acute promyelocytic leukemia cell line (HL-60), the human melanoma cell line (M14WM), the human breast adenocarcinoma cell line** (**MCF-7), and the human colon carcinoma (HT29) cell lines were purchased from American Type Culture Collection (Rockville, MD). Dulbecco's modified Eagle's medium (DMEM), Leibovitz's L-15 (L-15), penicillin, streptomycin, gentamicin, and L-glutamine were from GIBCO BRL; HEPES was from United States Biochemical; fetal bovine serum was from HyClone; the apoptosis inducers, well characterized and effective, for example, in disrupting mitochondrial transmembrane potential and activating caspases as well as inducing phosphatidyl-serine (PS) exposure, DNA fragmentation, and other apoptotic characteristics, were from PromoKine.

### 2.2. Plant Material

All plants used in the study* (Salvia officinalis, Malva sylvestris, Illicium verum, Glycyrrhiza glabra, Mentha piperita, Chamaemelum nobile, Melissa officinalis, Rhamnus Frangula, Thymus vulgaris, Plantago lanceolata, Calendula officinalis, Tilia europaea, Aloysia citrodora, Linum usitatissimum, Syzygium aromaticum, Coriandrum sativum, Cinnamomum verum, Papaver rhoeas, Aframomum, Helianthus annuus, Cuminum cyminum L., Sesamum indicum, Coffea arabica, Curcuma longa, Equisetum arvense, Hypericum perforatum, Origanum vulgare, Rosmarinus officinalis, Camellia sinensis, *and* Vaccinium myrtillus)* were purchased from a local herbalist in La Coruña and belonged to the pharmaceutical company Soria Natural, Soria, Spain. 50 grams of seeds, roots, or leaves was mechanically powered and extracted with 70% ethanol for 48 h at room temperature in a percolator. After this incubation extracts were filtered and evaporated with rotary evaporator to render the extracts alcohol free. All the extracts were weighted, collected in amber vials, and kept in refrigerator until being used.

### 2.3. Cell Culture

All cell lines were grown as a monolayer culture (except HL-60 which grows in suspension in RPMI 1640 medium) in DMEM (HS 5.T, HS 39T, MCF-7, and HT29), L15 (SW872, SW982), and MEM (M14WM) supplemented with 1% nonessential amino acids, 1% L-glutamine, 100 IU per mL penicillin, 100 IU per mL streptomycin, 20 mg/mL glutamine, and 10% fetal bovine serum (20% for HL-60 cell line) at 37°C in a 5% CO_2_ humidified atmosphere and 95% air in a CO_2_ incubator. Cells were passed twice/week under sterile conditions.

### 2.4. Cell Viability and Cell Morphology

Log phase cell suspension of all cell lines at a concentration of 10^5^/mL in culture medium (with 10% FBS) was used for the experiments in 96-well microtitre sterile plate. To each well 100 *μ*L of cell suspension was placed. The test extract was added at different concentrations (5, 25, and 50 *μ*g/mL) and the viable count was done by Trypan blue exclusion method after 24 h of treatment. To identify morphological changes induced by extract treatment, cells were examined using a phase contrast microscope. Cells displaying apoptotic changes were identified using previously defined morphological criteria including chromatin condensation, nuclear fragmentation, and blebbing of cytoplasmic membrane.

### 2.5. Antiproliferative Assay

Cell lines at exponential growth phase were washed, trypsinized, and resuspended in fresh medium. Cells were kept at a concentration of 10^5^ cells/well in 96-microtitre plate. The cells were treated with different concentration of test extract (5, 25, and 50 *μ*g/mL) against the apoptosis inducers as a positive controls (actinomycin D, dexamethasone, and etoposide, at a concentration of 1 *μ*g/mL/each) and the negative controls which contained only the medium and incubated for 24 h at 37°C. MTT [3-(4,5-dimethylthiazole-2-yl)-2,5-diphenyltetrazolium bromide] solution was added to each well to obtain the final concentration of 400 *μ*g/mL and further incubated at 37°C in a CO_2_ incubator for 3 h. The reaction resulted in the reduction of MTT by the mitochondrial dehydrogenase of viable cells to a purple formazan product. The MTT-formazan product was dissolved in DMSO and estimated by measuring the absorbance at 570 nm in an ELISA plate reader.

### 2.6. Apoptosis Quantification by ELISA

Apoptosis was determined by using a commercial cellular DNA fragmentation ELISA, a photometric enzyme-linked immunosorbent assay for the detection of BrdU-labeled DNA fragments in culture supernatants and cell lysates. Cells proliferating in vitro were incubated at 37°C in a humidified atmosphere (5% CO_2_) for 6 h with the nonradioactive thymidine analogue BrdU, which is incorporated into the genomic DNA. Plates were centrifuged 10 min at 250 ×g. Supernatants were removed and 100 *μ*L of lysing solution was added to wells and incubated for 30 min at room temperature. Plates were centrifuged and BrdU-labeled DNA fragments released from the cells into the cell cytoplasm during apoptosis were detected immunologically by using an anti-DNA antibody bound to the microtitre plate to capture the DNA fragments. After an incubation of 90 min at room temperature with anti-BrdU-antibody peroxidase-conjugate, plates were washed, 100 *μ*L of substrate solution was added to each well, and plates were read in an ELISA reader at 450 nm.

### 2.7. Reverse Transcriptase Polymerase Chain Reaction (RT-PCR)

Total RNAs were isolated from SW872, SW982, HS 39.T, HS 5.T, HL-60, M14WM, MCF-7, and HT29 cell lines treated with* Illicium verum, Glycyrrhiza glabra, Rhamnus Frangula, and Linum usitatissimum *extracts. Total RNAs were isolated according to the acid guanidium thiocyanate-phenol-chloroform extraction method [18]. RNA was precipitated in absolute cold alcohol, washed twice in 70% ethanol, dried, and resuspended in sterile water. Total RNA was quantified by NanoDrop™ Lite Spectrophotometer (Thermo Scientific, Waltham, MA, USA). One microgram of RNA was reverse-transcribed into cDNA by using a synthesis kit and oligo-dT (Clontech Labs, Palo Alto, CA). The resulting cDNA was amplified by PCR with the following set of primers: p53 [exon 7 (sense: TCT CCT AGG TTG GCT CTG ACT G; antisense: GCA AGT GGC TCC TGA CCT GGA)]; *β*-globin (sense: CTT CAT CCA CGT TCA CC; antisense: ACA CCT GTG TTC ACT AGC); Bcl-2 (sense: AGA TGT CCA GCC AGC TGC CAC CTG AC; antisense: TCG AAC GAG TGG GAC CAC GGA TAG A); Bcl-x (sense: CGG GCA TTC AGT GAC CTG AAC; antisense: GAA GTT GGC GAC CAA GGA CT); Bax (sense: ATG GAC GGG TCC GGG GAG CAG C; antisense: TTG TAC ATT TGG TAC ATC ACT CCA). Real time PCR reactions were performed using SybrGreen master mix in a 7500 real time PCR system (Applied Biosystems, Walthman, MA, USA). 35 cycles were carried out, each consisting of denaturation at 95°C for 30 sec, annealing for 30 sec at 60°C, and extension at 72°C for 30 sec. Extension during the final cycle continued at 72°C for 10 min. The resulting amplified fragments were identified on 1.5% agarose gel electrophoresis by ethidium bromide staining and the intensity of bands was quantified by densitometry. To calculate the changes in mRNA expression, we first normalized mRNA expression of target gene to the *β*-globin mRNA expression in a given sample; the mRNA expression for each gene in the experimental treatment was compared with the level of mRNA expression in the nontreated cells.

### 2.8. Statistical Analysis

Data were analyzed using SPSS 12.0 software for Windows. The experimental data were expressed as mean ± SD, the significance of difference among the various treated groups and positive and negative control groups was analyzed by means of one-way ANOVA, and the level of significance was set at *p* < 0.05.

## 3. Results

**Figure 1:**
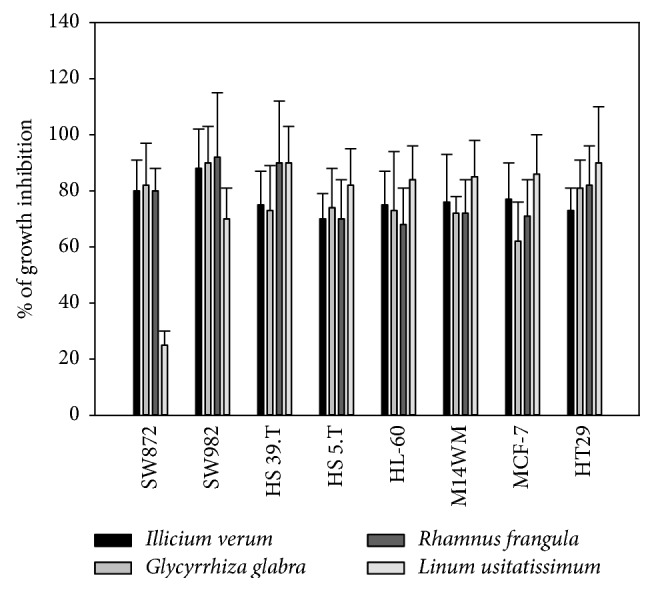
Effects of* Illicium verum *and* Linum usitatissimum *seed extracts*, Glycyrrhiza glabra *root extracts, and* Rhamnus Frangula* leave extracts on the growth of different tumor cell lines.

To demonstrate the potential biological activity of alcoholic extracts, we carried out a screening of the extracts by measuring their in vitro antiproliferative properties against several common human cancer cell lines. Some plant extracts exhibited antiproliferative activity toward all the cancer cell lines. The cellular growth inhibition percentages for the positive extracts are reported in Figure 1. The alcoholic extracts exhibiting the strongest antiproliferative activities in vitro at 50 *μ*g/mL concentration were* Illicium verum, Glycyrrhiza glabra, Rhamnus Frangula,* and* Linum usitatissimum*. Their levels of growth inhibition occurred at very similar values with regard to all the different cell lines. A weaker activity was observed for the* Linum usitatissimum*, which showed only a 25% of inhibition toward the SW872 cell line. The lowest in vitro inhibitory activity was observed for the* Mentha piperita, Chamaemelum nobile, Melissa officinalis, Thymus vulgaris, Plantago lanceolata, Calendula officinalis, Tilia europaea, Aloysia citrodora, Syzygium aromaticum, Coriandrum sativum, Cinnamomum verum, Papaver rhoeas, Aframomum, Helianthus annuus, Cuminum cyminum L., Sesamum indicum, Coffea arabica, Curcuma longa, Equisetum arvense, Hypericum perforatum, Origanum vulgare, Rosmarinus officinalis, Camellia sinensis, *and* Vaccinium myrtillus *plant extracts, all being inactive as antiproliferative agent toward the human cell lines (Table 1). The four alcoholic extracts exhibiting the most significant antiproliferative activities at 50 *μ*g/mL* (Illicium verum, Glycyrrhiza glabra, Rhamnus Frangula,* and* Linum usitatissimum)* were tested at lower concentration, 10 and 25 *μ*g/mL, respectively. Data are reported in Tables 2 and 3. Although the cellular growth inhibition percentages were reduced, they still show significant antiproliferative effects with respect to the untreated controls. The* Linum usitatissimum* exhibited, at 10 *μ*g/mL, high levels of cell growth inhibition toward almost all cell lines. Both* Illicium verum* and* Glycyrrhiza glabra* showed low levels of activity toward the SW872, SW982, HS 5.T, and HL-60 cell lines.

**Figure 2:**
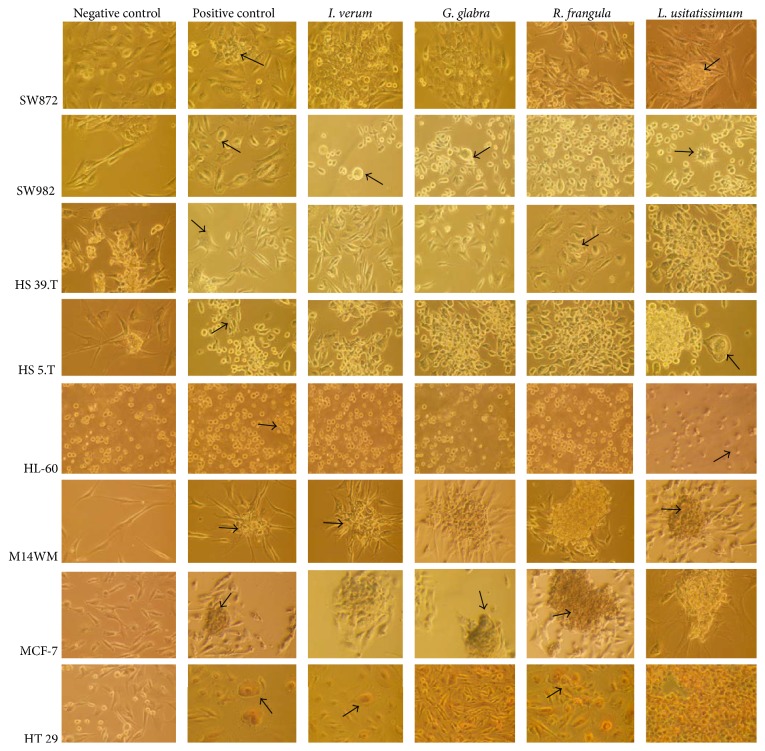
Phase contrast micrograph fields of untreated and treated cell lines used in the study. Black arrows show clear signs of apoptotic morphology (condensed/fragmented nuclei).

Drastic morphological changes, suggesting induction of apoptosis, were observed after treatment with* Illicium verum, Glycyrrhiza glabra, Rhamnus Frangula,* and* Linum usitatissimum *at a concentration of 25 *μ*g/mL. Representative results are shown in Figure 2. The untreated SW872 cells show only round and uniform cells growing in a monolayer shape. The positive SW872 cells treated with the apoptosis inducers show the characteristics features of apoptosis of small irregular nuclei, nuclear fragments, and red staining cytoplasm. In a similar way, SW872 cells treated with* Illicium verum *show signs of apoptosis (membrane blebbing, fragmented nuclei).

A significant level of apoptosis was observed in SW872, SW982, HS 39.T, HS 5.T, HL-60, M14WM, MCF-7, and HT29 cell lines treated with* Illicium verum, Glycyrrhiza glabra, Rhamnus Frangula,* and* Linum usitatissimum *at a concentration of 10, 25, and 50 *μ*g/mL, as compared to negative controls (Table 4). The highest apoptosis level was found in SW872, SW982, HS 39.T, HS 5.T, HL-60, M14WM, MCF-7, and HT29 cell lines treated with* Rhamnus Frangula *and no difference between extract's concentrations was observed, suggesting the presence of factors with a strongly apoptosis inducer activity at very low concentrations.

The major biochemical hallmark of apoptotic cell death is the cleavage of chromosomal DNA at internucleosomal sites into fragments or multiple of about 200 bp. DNA isolated from SW872, SW982, HS 39.T, HS 5.T, HL-60, M14WM, MCF-7, and HT29 cell lines treated with* Illicium verum, Glycyrrhiza glabra, Rhamnus Frangula,* and* Linum usitatissimum *at a concentration of 25 *μ*g/mL for two days showed DNA fragmentation patterns (data not shown).

**Figure 3:**
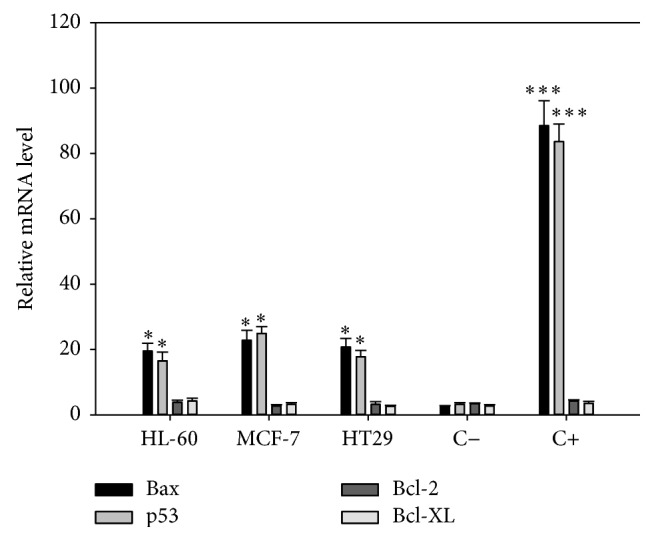
mRNA expression profile of Bax, p53, Bcl-2, and Bcl-XL in HL-60, MCF-7, and HT29 cell lines. A total of 1 × 10^6^ cells were treated with 10 *μ*g/mL of alcoholic extracts for 24 h. Total RNA was isolated and treated with DNase; 1 *μ*g of RNA was reverse-transcribed into cDNA with a synthesis kit, using oligo-dT and a random hexamer. mRNA levels were compared by RT-qPCR. Results were normalized to the *β*-globin gene and expressed as the mean ± SD relative to the negative control (C−, untreated cells). As positive control (C+) cells were treated with 7 *μ*M of staurosporine. Experiments were done in triplicate. ^*∗*^*p* < 0.05 versus C−; ^*∗∗∗*^*p* < 0.001 versus C−.

RT-PCR analysis detected Bax and p53 mRNAs in almost all cell lines treated with 10 *μ*g/mL of* Illicium verum, Glycyrrhiza glabra, Rhamnus Frangula,* and* Linum usitatissimum*. The percentage of positive mRNA expression was variable for different cell lines, being the highest positive cell lines observed in the group treated with* Glycyrrhiza glabra *and* Rhamnus Frangula*. Conversely, the expression of both Bcl-2 and Bcl-XL was almost negative in all cell lines treated with* Illicium verum, Glycyrrhiza glabra, Rhamnus Frangula,* and* Linum usitatissimum* extracts (Figure 3). Since RT-PCR is a very sensitive method to detect minimal levels of RNA, it is reasonable to think that both Bcl-2 and Bcl-XL are completely switched off in treated cell lines.

## 4. Discussion

Shikimic acid extracted from the pods (which wraps the seeds) of Chinese star anise* (Illicium verum)* is the starting material of Tamiflu® (Roche Laboratories), an antiviral drug which has gained popularity with the recent spread of the bird flu (H5N1). In addition anise extract typically contains 1% to 3% volatile anise oil. The primary constituent of anise oil is anethole (80% to 90%). Other components include alpha-pinene, linalool, anisaldehyde, and methyl chavicol. The composition of anise oil from* Illicium verum* resembles that of anise oil obtained from* Pimpinella anisum* but also contains trace quantities of safrole and myristicin [19].

*Glycyrrhiza glabra* (licorice) has a long history of medicinal use in Europe and Asia. At high doses, it shows potentially severe side effects, including hypertension (high blood pressure), hypokalemia (low blood potassium levels), and fluid retention. Most adverse effects have been attributed to triterpene saponins (glycyrrhiza, or glycyrrhizic, constitutes the major chemical components of licorice). The other compounds vary from species to species and depend on the provenance of the plant. Several flavonoids, as well as other phenolic constituents, are found in licorice; amines, amino acids, sterols, sugars, and starch are also present [20] and ethanolic extract of* G. glabra* has shown considerable antioxidant activity and protective effect against the human lipoprotein oxidative system.

A systematic fractionation of an ethanol-water (1 : 1) extract of the seeds of* Rhamnus Frangula*, guided by assays for tumor-inhibitory activity, led to the isolation of aloe emodin [21]. This compound was found to show significant antileukemic activity against the P-388 lymphocytic leukemia in mice [22].

*Linum usitatissimum* is a rich source of the essential fatty acid alpha-linolenic acid, which is a biologic precursor to omega-3 fatty acids such as eicosapentaenoic acid. Although omega-3 fatty acids have been associated with improved cardiovascular outcomes, evidence from human trials is mixed regarding the efficacy of flaxseed products for coronary artery disease or hyperlipidemia. The lignan constituents of flaxseed (not flaxseed oil) possess in vitro antioxidant and possible estrogen receptor agonist/antagonist properties, prompting theories of efficacy for the treatment of breast cancer.

The results obtained in this study suggest that alcoholic extracts from* Illicium verum, Glycyrrhiza glabra*,* Rhamnus Frangula,* and* Linum usitatissimum* are able to induce growth inhibition and apoptosis and modulate the expression of both Bax and p53 proapoptotic genes and the extent of gene expression was obtained at only 10 *μ*g/mL cell culture treatment. Although with some differences, all tumor cell lines showed similar results, being the highest regulation in gene expression observed in SW 982 and SW 872 cell lines with low differences between the four alcoholic extracts.

These results are in line with previous reports that suggested a possible use for the management of metastatic malignant tumors [23]. In a recent study RT-PCR results showed downregulation of HSP90 gene expression which implied an ability of* Glycyrrhiza glabra* to induce apoptosis in HT-29 cells and confirmed its anticancer property [24]. In addition, the extract of* Linum usitatissimum *containing mainly sterols and triterpenes (21.4%), free fatty acids (17.8%), lignin dimers (12.2%), and lipids (7.7%) showed significant cell lethality and suppression of cell vitality and proliferation of tumor cell lines [25].

Many advances in the management of cancer have been achieved since those early times when cancer chemotherapy started to take shape with the discovery of antitumor activity of alkylating agents [26] and certain hormones [27] and the advent of antimetabolites of DNA building blocks. In the Sixties and later, clinical studies of combination therapy brought about major advances leading to the demonstration that complete remission of certain neoplasia could be induced with available anticancer drugs. At the same time, new agents such as anthracycline antibiotics [28], the Vinca alkaloids [29], the Platinum complexes [30], and hormone antagonists provided additional powerful tools [31].

The advances in cancer chemotherapy were also greatly aided by progress made in diagnostic procedures, by the advent of combined modalities of treatment, and, last but not least, by the development of improved criteria for regimen design and result assessment [32]. As a consequence of these advances, it is now possible to induce complete tumor regression in patients with different types of neoplasia and to obtain disease-free survival lusting many years in a significant percentage of them.

Despite this progress, major difficulties remain to be overcome before cancer therapeutics can become generally successful in the curative management of cancer. This is particularly so in the case of the common so-called solid tumors. These difficulties can be attributed for the most part to the lack of agents acting uniquely and specifically on tumors, or at least having sufficiently marked selectivity of antitumor action, and to the phenomenon of resistance.

These two limitations combined are the main reasons for ultimate failure: in fact, even a minor level of cellular resistance may be sufficient to impart clinical resistance because one cannot overcome it by increasing adequately drug dose intensity without incurring nonacceptable toxicity. During the past two decades new vistas have been opening up in cancer therapeutics consequent to progress made in the understanding of the molecular biology of the cancer cell, of the interaction between tumor and host regulatory mechanisms, and of the mechanisms responsible for different forms of resistance.

The main purpose of this study was to evaluate the induction of apoptosis in human cell lines treated with alcoholic herbal extracts. Apoptosis is a selective physiologic deletion of cells that are no longer required. The concept of apoptosis could be of great importance for cancer treatment since the internal cellular program of cells to commit suicide can be initiated by cell damaging agents like antineoplastic drugs as well as by natural derived compounds. Some researchers have postulated that the effect of some antineoplastic compounds is caused by apoptosis-induction. It has been found by several authors that inhibition of apoptosis induces resistance to chemotherapeutic drugs [33]. Bax and p53 are the most well-known proapoptotic genes, and both are important in multicellular organisms, where they regulate the cell cycle and thus function as a tumor suppressor that is involved in preventing tumor development. Current studies about the interrelationship between drug resistance and apoptosis were mainly determined by Bax and p53 and therefore for the intrinsic power to inhibit apoptosis [34].

Although much remains to be learned in each of antitumor research areas that no doubt will have an impact on the management of cancer, it is already possible to identify new directions in cancer therapeutics aimed at exploiting the knowledge acquired so far. The approaches that are being pursued at present are essentially as follows: (1) the development of specific or highly selective antitumor agents affecting newly discovered cellular sites of potential intervention; (2) the increase of antitumor effectiveness of available drugs through the design of improved treatments, including combination treatments based on the knowledge of the mechanisms of antitumor and toxic drug action; (3) the modification or prevention of resistance to individual or multiple drugs, whether natural or acquired, through the alteration of relevant gene expression and/or the modification of biochemical mechanisms involved; (4) the induction of therapeutically profitable alterations of biological responses to tumor through the administration of soluble or cellular effectors, or their blockage and/or modulation; (5) the development of combined treatment modalities maximizing antitumor action and taking advantage of differences in the limiting toxicities of the treatments combined; and (6) the development of treatments tailored to individual patients based on improved assessments of potential tumor responsiveness and of the limitations related to the heterogeneity of tumor cell populations.

In summary, extracts of* Illicium verum, Glycyrrhiza glabra, Rhamnus Frangula,* and* Linum usitatissimum* have shown significant reduction in in vitro tumor cell proliferation. Additional studies are needed to identify how these natural extracts could be used as a complementary approach to currently used chemotherapies for different cancers. Also, more studies designed to investigate the molecular mechanisms underlying anticancer activity are needed. Altogether, natural extract use holds promise as an adjuvant treatment to prevent tumor cell growth.

## Figures and Tables

**Table 1:** Growth inhibition percentages recorded at screening concentration of 50 *μ*g/mL.

	SW872	SW982	HS 39.T	HS 5.T	HL-60	M14WM	MCF-7	HT29
*Salvia officinalis*	8	11	10	11	9	8	9	9
*Malva sylvestris *	7	10	11	9	8	7	3	10
*Mentha piperita *	8	11	9	11	9	8	6	9
*Chamaemelum nobile *	9	10	9	8	9	7	8	5
*Melissa officinalis *	10	11	10	11	9	7	9	6
*Thymus vulgaris *	11	11	12	11	12	13	12	9
*Plantago lanceolata *	9	9	11	13	14	11	9	6
*Calendula officinalis *	8	11	12	11	14	11	10	8
*Tilia europaea *	5	9	11	12	11	10	8	5
*Aloysia citrodora *	7	11	11	10	14	11	5	6
*Syzygium aromaticum *	8	11	11	15	14	11	7	9
*Coriandrum sativum *	10	11	10	12	13	10	11	9
*Cinnamomum verum *	11	10	9	7	6	8	11	10
*Papaver rhoeas *	15	11	12	11	10	11	10	5
*Aframomum *	10	10	11	12	11	15	11	6
*Helianthus annuus *	11	11	12	11	9	8	6	4
*Cuminum cyminum* L.	9	6	7	7	4	9	11	10
*Sesamum indicum *	8	11	12	11	15	11	13	11
*Coffea arabica *	8	12	13	11	11	10	11	12
*Curcuma longa *	9	9	9	7	7	8	10	10
*Equisetum arvense *	7	11	12	11	13	11	10	10
*Hypericum perforatum *	7	9	9	8	9	7	7	12
*Origanum vulgare *	10	11	12	13	11	10	9	9
*Rosmarinus officinalis *	9	11	10	11	11	13	15	15
*Camellia sinensis *	4	11	10	14	15	15	15	11
*Vaccinium myrtillus *	8	12	11	14	9	9	11	9

Values are the mean of at least three independent determinations; coefficient of variation was less than 15%; not significant (below 15% inhibition).

**Table 2:** Growth inhibition percentages recorded at screening concentration of 25 *μ*g/mL.

	SW872	SW982	HS 39.T	HS 5.T	HL-60	M14WM	MCF-7	HT29
*Illicium verum*	65 ± 9	70 ± 15	70 ± 14	65 ± 13	60 ± 12	66 ± 13	61 ± 19	58 ± 16
*Glycyrrhiza glabra*	70 ± 22	75 ± 14	70 ± 18	68 ± 13	65 ± 15	65 ± 21	55 ± 16	58 ± 18
*Rhamnus frangula*	65 ± 13	71 ± 23	72 ± 12	60 ± 16	61 ± 15	66 ± 16	60 ± 17	60 ± 18
*Linum usitatissimum*	20 ± 6	58 ± 18	65 ± 10	66 ± 17	60 ± 14	55 ± 19	58 ± 16	65 ± 13

Values are the mean of at least three independent determinations; coefficient of variation was less than 15%; not significant (below 15% inhibition).

**Table 3:** Growth inhibition percentages recorded at screening concentration of 10 *μ*g/mL.

	SW872	SW982	HS 39.T	HS 5.T	HL-60	M14WM	MCF-7	HT29
*Illicium verum *	13 ± 8	14 ± 3	12 ± 4	11 ± 4	14 ± 9	28 ± 11	31 ± 11	25 ± 8
*Glycyrrhiza glabra *	11 ± 8	11 ± 7	21 ± 8	13 ± 7	12 ± 5	27 ± 9	11 ± 4	28 ± 4
*Rhamnus frangula *	11 ± 6	14 ± 3	26 ± 9	11 ± 6	32 ± 12	25 ± 5	12 ± 4	12 ± 8
*Linum usitatissimum *	19 ± 6	25 ± 11	25 ± 6	31 ± 7	26 ± 7	28 ± 8	26 ± 4	23 ± 7

Values are the mean of at least three independent determinations; coefficient of variation was less than 15%; not significant (below 15% inhibition).

**Table 4:** Induction of apoptosis by 10, 25, and 50 *μ*g/mL of *Illicium verum, Glycyrrhiza glabra, Rhamnus Frangula, *and* Linum usitatissimum *alcoholic extracts in SW872, SW982, HS 39.T, HS 5.T, HL-60, M14WM, MCF-7, and HT29 cell lines measured by ELISA.

	SW872	SW982	HS 39.T	HS 5.T	HL-60	M14WM	MCF-7	HT29
*Illicium verum, *10 *μ*g/mL	45 ± 4	55 ± 11	54 ± 11	26 ± 5	33 ± 8	32 ± 10	17 ± 10	22 ± 7
*Illicium verum, *25 *μ*g/mL	66 ± 5	57 ± 10	45 ± 13	33 ± 9	36 ± 6	34 ± 12	26 ± 18	28 ± 9
*Illicium verum, *50 *μ*g/mL	70 ± 5	67 ± 12	58 ± 14	45 ± 9	44 ± 8	45 ± 16	38 ± 13	25 ± 10
*Glycyrrhiza glabra, *10 *μ*g/mL	55 ± 11	44 ± 9	55 ± 10	26 ± 11	46 ± 10	43 ± 8	44 ± 9	28 ± 7
*Glycyrrhiza glabra, *25 *μ*g/mL	67 ± 12	45 ± 7	58 ± 11	32 ± 12	47 ± 11	44 ± 11	57 ± 9	28 ± 9
*Glycyrrhiza glabra, *50 *μ*g/mL	65 ± 11	66 ± 11	77 ± 12	35 ± 11	44 ± 9	44 ± 15	88 ± 23	33 ± 9
*Rhamnus frangula, *10 *μ*g/mL	66 ± 9	77 ± 11	65 ± 14	75 ± 12	65 ± 12	88 ± 9	78 ± 9	74 ± 11
*Rhamnus frangula, *25 *μ*g/mL	70 ± 11	74 ± 12	64 ± 11	78 ± 21	68 ± 15	62 ± 12	68 ± 11	75 ± 21
*Rhamnus frangula, *50 *μ*g/mL	78 ± 9	88 ± 11	79 ± 13	89 ± 22	98 ± 17	96 ± 10	95 ± 12	86 ± 24
*Linum usitatissimum, *10 *μ*g/mL	43 ± 10	54 ± 10	34 ± 12	34 ± 11	32 ± 11	19 ± 6	44 ± 13	18 ± 6
*Linum usitatissimum, *25 *μ*g/mL	56 ± 12	77 ± 14	44 ± 16	45 ± 9	46 ± 10	25 ± 11	58 ± 14	34 ± 9
*Linum usitatissimum, *50 *μ*g/mL	50 ± 14	86 ± 18	54 ± 18	55 ± 9	66 ± 13	28 ± 11	98 ± 28	66 ± 16

The data represent mean ± SD of at least three independent experiments; coefficient of variation was less than 15%; data are expressed as the mean percentage of apoptotic cells.
